# Theaflavin-3,3′-Digallate Promotes the Formation of Osteoblasts Under Inflammatory Environment and Increases the Bone Mass of Ovariectomized Mice

**DOI:** 10.3389/fphar.2021.648969

**Published:** 2021-03-23

**Authors:** Gaoran Ge, Sen Yang, Zhenyang Hou, Minfeng Gan, Huaqiang Tao, Wei Zhang, Wenming Li, Zheng Wang, Yuefeng Hao, Ye Gu, Dechun Geng

**Affiliations:** ^1^ Department of Orthopaedics, The First Affiliated Hospital of Soochow University, Suzhou, China; ^2^ Suzhou Ninth People’s Hospital, Suzhou Ninth Hospital affiliated to Soochow University, Suzhou, China; ^3^ Department of Orthopaedics, Teng Zhou Central People’s Hospital, Tengzhou Hospital Affiliated to Xuzhou Medical University, Tengzhou, China; ^4^ Department of Orthopaedics, Suzhou Kowloon Hospital, Shanghai Jiao Tong University School of Medicine, Suzhou, China; ^5^ Orthopedics and Sports Medicine Center, Suzhou Municipal Hospital (North District), Nanjing Medical University Affiliated Suzhou Hospital, Suzhou, China; ^6^ Department of Orthopaedics, Changshu Hospital Affiliated to Soochow University, First People’s Hospital of Changshu City, Changshu, China

**Keywords:** osteoblast, bone formation, osteoprosis, inflammatory cyotokines, TFDG

## Abstract

Postmenopausal osteoporosis is a disease of bone mass reduction and structural changes due to estrogen deficiency, which can eventually lead to increased pain and fracture risk. Chronic inflammatory microenvironment leading to the decreased activation of osteoblasts and inhibition of bone formation is an important pathological factor that leads to osteoporosis. Theaflavin-3,3′-digallate (TFDG) is an extract of black tea, which has potential anti-inflammatory and antiviral effects. In our study, we found that TFDG significantly increased the bone mass of ovariectomized (OVX) mice by micro-CT analysis. Compared with OVX mice, TFDG reduced the release of proinflammatory cytokines and increased the expression of osteogenic markers *in vivo. In vitro* experiments demonstrated that TFDG could promote the formation of osteoblasts in inflammatory environment and enhance their mineralization ability. In this process, TFDG activated MAPK, Wnt/β-Catenin and BMP/Smad signaling pathways inhibited by TNF-α, and then promoted the transcription of osteogenic related factors including Runx2 and Osterix, promoting the differentiation and maturation of osteoblasts eventually. In general, our study confirmed that TFDG was able to promote osteoblast differentiation under inflammatory environment, enhance its mineralization ability, and ultimately increase bone mass in ovariectomized mice. These results suggested that TFDG might have the potential to be a more effective treatment of postmenopausal osteoporosis.

## Introduction

Postmenopausal osteoporosis is a disorder of decreased bone mass, microarchitectural deterioration, and fragility fractures, which affects more than 200 million women worldwide (Ma et al., 2020). It often occurs 5–10 years after menopause. With the growth of female population and the prolongation of people’s life span, the prevalence of postmenopausal osteoporosis will continue to increase. At present, the clinical treatment of postmenopausal osteoporosis is usually hormone replacement therapy, calcium, vitamin D, and other methods (You and Liu, 2020). However, these are usually not be satisfied due to the endocrine disorders, or delayed onset of effect and other factors (Walallawita et al., 2020). Therefore, it is urgent to find a more effective treatment drug.

Bone is a kind of tissue that forms and degrades continuously. This remodeling is a tightly controlled process and may be disturbed by many factors. Progressive bone loss and destruction is a major pathological feature of chronic inflammatory disease, including postmenopausal osteoporosis. Chronic inflammation can disrupt bone metabolism and inhibit bone formation (Straub et al., 2015). The decrease of estrogen level is the basic mechanism of bone loss in postmenopausal women (Sapir-Koren and Livshits, 2017). Estrogen levels in normal women protect the bones from the negative balance of bone metabolism due to increased bone turnover. However, with the advent of perimenopause and premature menopause, the rapid decline or stop of ovarian function makes the level of estrogen drop suddenly. The decrease of estrogen promotes the release of proinflammatory cytokines including interleukin-1 (IL-1), IL-6 and tumor necrosis factor α (TNF-α), which hinders the activation of osteoblasts and reduces bone formation (Pacifici, 1996). Osteoblasts are the main functional cells of bone formation, responsible for the synthesis, secretion and mineralization of bone matrix (Hu and Olsen, 2016). It has been known that osteoblasts are rich in alkaline phosphatase (ALP) and are generated from the osteogenic differentiation of mesenchymal stem cells (Papachroni et al., 2009). In the process of bone formation, hormones and cytokines combined with the corresponding receptors can activate a variety of intracellular signal transduction pathways, thus promoting the differentiation of osteoblasts (Schmid, 1993). During this process, mitogen activated protein kinase (MAPK), Wnt/β-catenin and bone morphogenetic protein (BMP)/Smad signaling pathways play important roles in the regulation of osteogenesis (Chen et al., 2012; Yin et al., 2018; Kim et al., 2019). The activation of these signaling pathways, on the one hand, can start and activate osteoblast specific transcription factors [Runt-related transcription factor 2 (Runx2), Osterix], thus inducing the differentiation of osteoblasts (Han et al., 2017; Komori, 2019); on the other hand, they can participate in the regulation of cell division and promote the cells to stop division and turn to osteoblast differentiation (Liu et al., 2019). However, in the chronic inflammatory microenvironment of osteoporosis, proinflammatory cytokines block the activation of osteoblast signaling pathway, inhibit the formation of osteoblasts, and ultimately reduce bone formation (Liu et al., 2016). Therefore, how to promote the formation of osteoblasts is the key to reverse osteopenia in postmenopausal osteoporosis.

Traditional Chinese medicine is an important part of Chinese culture, and the effective ingredients extracted from traditional Chinese medicine have been used to treat a variety of clinical diseases (Wang et al., 2020). Theaflavin-3,3′-digallate (TFDG) is a class of polyphenol hydroxyl compounds with the structure of tea polyphenol ketone, which is extracted from dicotyledon tea [*Camellia sinensis* (L.) O. Kuntze.]. It has been reported that TFDG plays an important role in regulating blood lipid, preventing cardiovascular disease and antivirus (Isaacs and Xu, 2013; Shen et al., 2019). Notably, studies have shown that TFDG can inhibit macrophage production of nitric oxide, down-regulate NF-κB activation, thus attenuating inflammatory response eventually (Ukil et al., 2006). In addition to these findings, however, the role of TFDG on osteoblastogenesis in the model of postmenopausal osteoporosis is still unclear. In this study, we found that TFDG significantly increased the bone mass in ovariectomized (OVX) mice and it was able to enhance the osteoblast formation ability, which showed an osteoprotective effect of TFDG and might bring a new potential treatment for postmenopausal osteoporosis.

## Materials and Methods

### Animal Models

All experiments were conducted according to the Ethics Committee of the First Affiliated Hospital of Soochow University and the Guidelines for Care and Use of Laboratory Animals (201910A298). 32 female C57BL/6 mice (8–10 weeks) were randomly divided into 4 groups: sham control [healthy mice treated with phosphate buffered saline (PBS), *n* = 8], vehicle (OVX mice treated with PBS, *n* = 8), low TFDG (MedChemExpress, New Jersey, United States) treated group (1 mg/kg, *n* = 8), high TFDG treated group (10 mg/kg, *n* = 8). Bilateral ovariectomy was carried out to induce osteoporosis under pentobarbital sodium (Sigma-Aldrich, St. Louis, United States) anesthesia for the mice in OVX group and OVX + TFDG groups, a sham procedure in which the ovaries were only exteriorized but not resected was performed for the mice in sham group. After 5 weeks intervention, all animals were sacrificed. Serum was collected and stored at −80°C prior to biomarker assay, while the bilateral femurs were collected for histological and immunohistochemical analysis, and micro-CT scanning.

### Micro-CT Analysis

Micro-CT (μCT) scan was conducted using a SkyScan 1176scanner (SkyScan, Aartselaar, Belgium). Briefly, the samples were placed in scanning warehouse. The parameters are set as follows: voltage 50 kV, current 800 μA, scanning range 2 × 2 cm, and scanning layer thickness 8 μm. The scan data were then entered into the computer to conduct three-dimensional reconstruction with NRecon software, and the bone tissue parameters were analyzed with CTAn software after reconstruction.

### Histology and Immunohistochemistry Staining

Tissues were collected and fixed with 10% formalin for 48 h. Bone tissues were decalcified in 10% ethylenediaminetetraacetic acid (EDTA, Sigma-Aldrich) for 4 weeks. The tissues were then dehydrated and paraffin embedded. Five-micrometer (5 mm) quadrate sections of the tissues were cut in the sagittal position on a microtome. H&E staining were performed to visualize histomorphology. The tissue sections were placed into xylene to dissolve the wax. The sections were then rehydrated in an ethanol solution. After being washed, the sections were immersed in hematoxylin dye (Leagene, Beijing, China) for 3 min. Color separation with 1% hydrochloric acid in ethanol and ammonia was then performed. The sections were then immersed in eosin dye (Leagene) and dehydrated with ethanol and xylene after being washed. For IHC staining, tissue sections were prepared for antigen restoration by immersion in 5% hydrogen peroxidase at 37°C for 10 min, after which they were incubated with primary antibodies for inflammatory factors including TNF-α (1:500), IL-10 (1:500), IL-1β (1:500), IL-6 (1:600), and osteogenic index including osteocalcin (OCN, 1:500), and Runx2 (1:600; all from Abcam, Cambridge, United Kingdom) over night at 4°C. Secondary antibodies (VECTOR, Burlingame, CA, United States) were utilized to combine with the primary antibodies according to the host of primary antibodies. Diaminobezidin (DAB, VECTOR) staining was then conducted to color the positive cells. Microscopic images were acquired using an inverted light microscope (OLYMPUS, Japan). The quantitative analysis was performed using Image J software.

### Measurement of Serum Marker Levels

The concentrations of cytokines in the sera of mice were measured using sandwich ELISA kits (Multi Sciences, Hangzhou, China), according to the manufacturer’s instructions.

### Cell Culture

Commercial mouse pre-osteoblastic MC3T3-E1 cells (Riken Cell Bank, Tsukuba, Japan) were used in this experiment. Cells were extracted from the long bones of the lower limbs and cultured in α-minimum essential medium (MEM; Hyclone, California, United States) containing 10% fetal bovine serum (FBS; Gibco, California, United States), and 100 U/ml penicillin/streptomycin (P/S, NCM Biotech, Suzhou, China). All cells were cultured in a 37°C standard environment and stored at −80°C with serum-free cell freezing medium (NCM Biotech).

### Cell Viability Assay

Cell survival rate was measured using the Cell Counting Kit 8 (CCK-8, ApexBio, Houston, United States). Briefly, cells (5 × 10^3^/well) were cultivated in a 96-well plate. Cells were treated with different concentrations of TFDG (0, 0.01, 0.1, 1, 10, and 20 μM) for 1, 4, and 7 days. After that, 10 μl CCK-8 assay solution was supplemented to 100 μl medium in every well. Then, cells were incubated for 2 h at 37°C. The optical density of every well was got on a microplate reader (BioTek, Vermont, United States). The inhibition rate was calculated as follows: inhibition rate (x) = (OD_control_−OD_x_)/OD_control._

### Alkaline Phosphatase Staining

MC3T3-E1 cells were cultured in osteogenic medium for 1 week with different stimulus. After fixation of cells in 4% paraformaldehyde for 15 min, the cells were washed three times with PBS and consequently dipped in BCIP/NBT working solution in the dark for 20 min. The staining outcomes were obtained under a microscope (OLYMPUS).

### Alizarin Red S Staining

Briefly, MC3T3-E1 cells were cultured in osteogenic medium for 3 weeks with different stimulus. We washed MC3T3-E1 cells for three times with PBS after 3 weeks induction in osteogenic medium. Then all cells were fixed in 4% paraformaldehyde for 20 min at 4°C. After that, the cells were washed with PBS and incubated in alizarin red S (ARS) staining solution (pH4.2; Solarbio, Beijing, China) for 20 min. At last, washed cells for three times. Microscopic images were acquired using an inverted light microscope (OLYMPUS).

### Immunofluorescence Staining

After culture, the medium was removed and MC3T3-E1 cells were fixed by 4% paraformaldehyde (NCM Biotech). 0.1% Triton X-100 (NCM Biotech) was used to permeabilize the cells for 10 min. In a next step, 10% bovine serum albumin (BSA) was employed to block the non-specific binding sites. Corresponding fluorochrome-conjugated secondary antibodies (goat anti-rat IgG H&L, Alexa Fluor 488, 1:1,000, Abcam; goat anti-rabbit IgG H&L Alexa Fluor 647, 1:1,000, Abcam) were used to combined with primary antibodies, and 4′, 6-diamidino-2-phenylindole (DAPI, 1:20; Yuanye, Shanghai, China) was stained for 10 min at room temperature. The cells were observed under a laser confocal microscope (Leica, TCS SP8, Germany).

### Real-Time PCR

Total mRNA was extracted using Beyozol reagent (Beyotime, Shanghai, China). The concentration and purity of mRNA were assessed by NanoDrop-2000 (Thermo Fisher, United States). PrimeScript RT Master Mix (Takara, Dalian, Japan) was used for reverse transcription. For PCR amplification, a total of 2 μl cDNA product was used for subsequent RT-qPCR analysis using SYBR1 Premix Ex Taq^TM^ (Takara). The reaction system includes 10 μl of Forget-Me-Not qPCR Master Mix (Biotium, United States), 1 μl primer, and 7 μl RNase Free H_2_O as well. CFX96 Touch Real-Time PCR Detection System (Bio-Rad, United States) was utilized for reaction process. The following are primer sequences for ALP, OCN, and Osterix (Supplementary Table S1).

### Western Blot

Cells were seeded in 6-well plates with a density of 1 × 10^6^ cells per well. The medium was then replaced by α-MEM with or without TNF-α or TFDG. Proteins were obtained by centrifugation (12,000 rpm) for 15 min at 4°C. Protein concentration was measured by BCA assay (Beyotime). Total protein was mixed with 5× loading buffer (Beyotime) and boiled at 95°C for 10 min. The proteins were separated by SDS polyacrylamide gel electrophoresis (SDS-PAGE; EpiZyme, Shanghai, China). Electrophoresis was performed using Bio-Rad (California, United States) equipment at 180 V for 40 min. Then, the proteins were transferred to a nitrocellulose membrane at 350 mA for 70 min using membrane transfer equipment (Bio-Rad). Put the membranes into the western block solution and block it for 1 h at room temperature. Then the membranes were incubated over night at 4°C with primary antibodies against ALP (1:1,000), Osterix (1:500), Runx2 (1:1000), OCN (1:500), ERK (1:1,000), p-ERK (1:1,000), β-catenin (1:1,000), Smad (1:1,000), p-Smad (1:1,000), and β-actin (1:1,000; Abcam). Then, membranes were rinsed in Tris-buffered saline with Tween 20 and incubated with corresponding secondary horse radish peroxidase-conjugated antibodies (1:5,000) for 2 h at room temperature. The proteins were detected employing a chemiluminescence detection system (Bio-Rad).

### Statistical Analysis

All data are expressed as the mean ± SD and assessed statistically using a one-way analysis of variance (one-way ANOVA) and an unpaired two-tailed Student’s *t*-test. A *p*-value < 0.05 was considered significant.

## Results

### TFDG Increased Bone Mass and Promoted Bone Formation of Trabeculae in OVX-Induced Osteoporosis Mice

**FIGURE 1:**
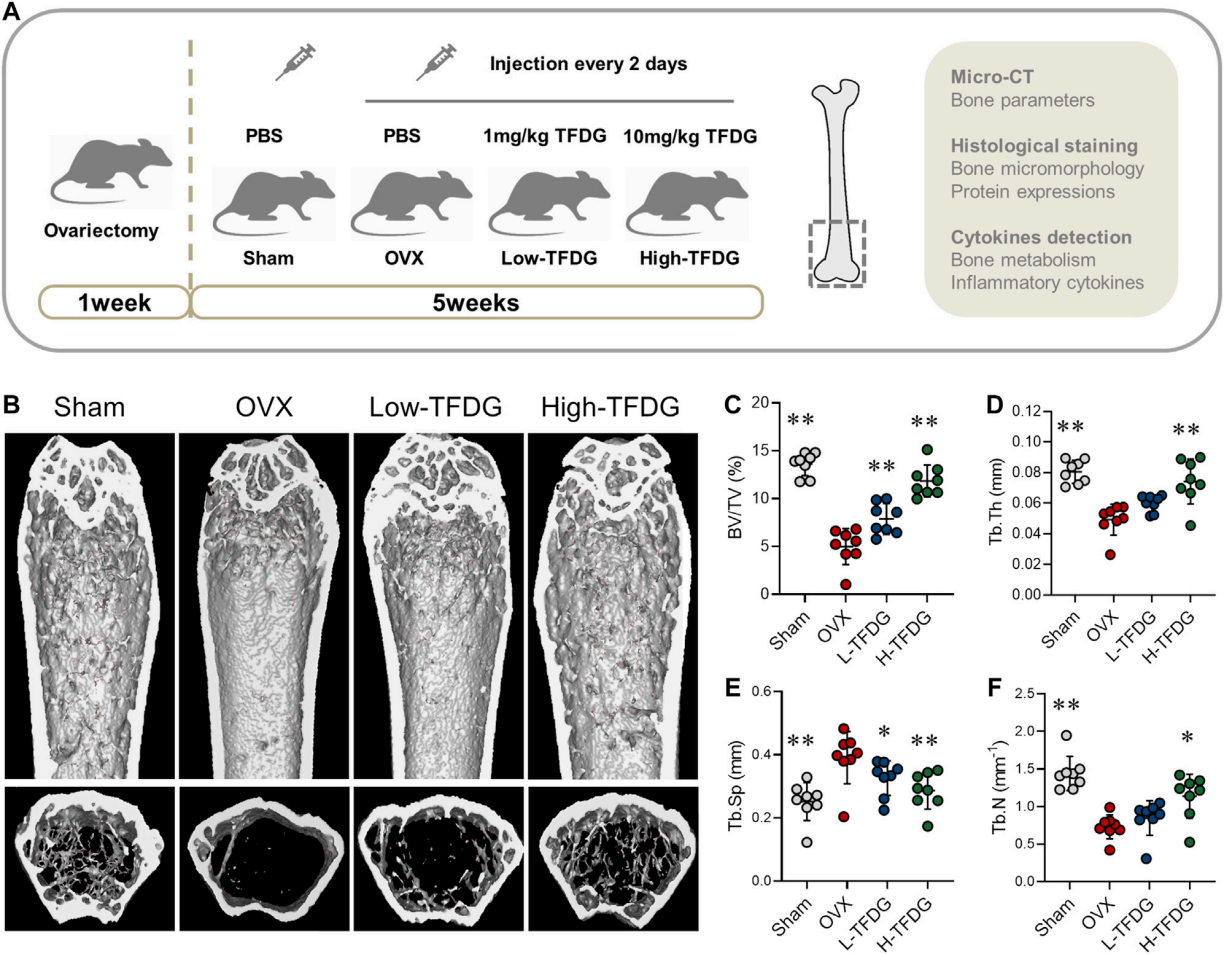
TFDG increased bone mass of OVX mice. **(A)** Schematic diagram of *in vivo* experiments. **(B)** Micro-CT reconstruction images. **(C)** Bone volume/tissue volume (BV/TV, %), **(D)** Trabecular thickness (Tb.Th, mm), **(E)** Trabecular separation (Tb.Sp, mm), **(F)** Trabecular number (Tb.N, mm^−1^). *n* = 8, *p < 0.05, **p < 0.01, compared with OVX group.

We used the OVX mice to investigate the effect of TFDG on the estrogen deficiency–induced bone loss. After ovariectomy, PBS and different concentrations of TFDG dilutions were injected every 2 days (Figure 1A). The related parameters of cancellous bone of distal femur in mice were detected and analyzed by Micro–CT. The results of three-dimensional reconstruction showed that there was obvious loss of bone trabeculae in the transverse section of distal femur in OVX group compared with Sham group. The destruction of bone trabeculae in the medullary cavity was particularly obvious. TFDG treatment increased the density of trabecular bone significantly (Figure 1B). The statistical results showed that the bone volume fraction (BV/TV), the average trabecular thickness (Tb.Th), trabecular spacing (Tb.Sp) and trabecular number (Tb.N) in the OVX group were much lower than those in the Sham group, and the average distance between the bone trabeculae in the OVX group was significantly higher. When treated with TFDG for 5 weeks, it was found that the cancellous bone at the distal end of the femur increased. At the same time, the thickness and number of trabeculae increased significantly, while the trabecular spacing decreased as well (Figures 1C–F).

**FIGURE 2:**
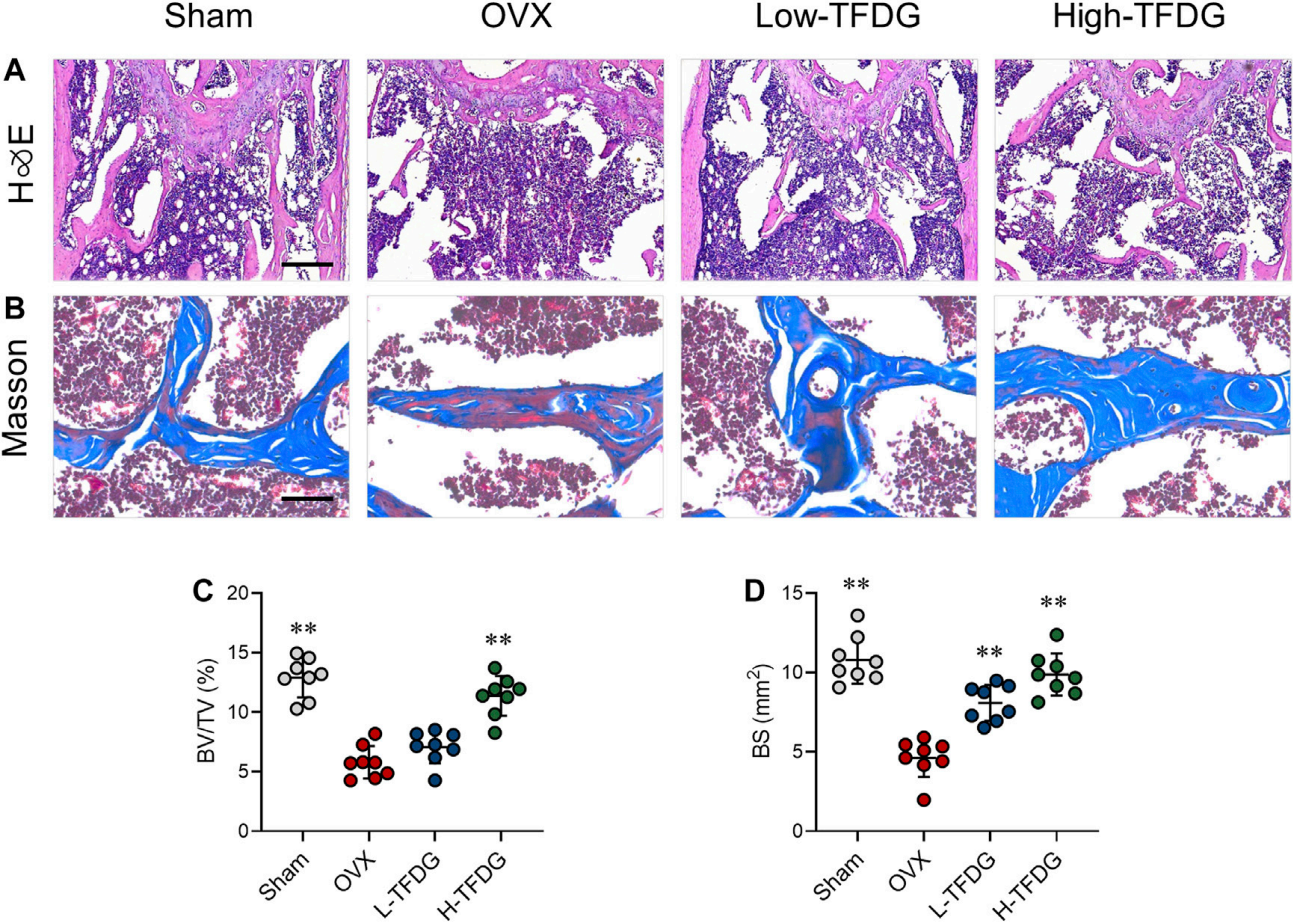
TFDG promoted trabecular bone formation in OVX mice. **(A)** H&E staining of bone tissue. Scale bar = 100 μm. **(B)** Masson staining of bone tissue (blue). Scale bar = 50 μm. **(C)** Quantitative analyses of histomorphometric bone parameters of BV/TV (%) and **(D)** bone surface area (BS mm^2^). *n* = 8, **p < 0.01, compared with OVX group.

In order to observe the microstructure changes of cancellous bone, H&E, and Masson staining were conducted. Compared with sham group, the number and thickness of trabeculae in OVX mice were significantly decreased. After TFDG treatment, the bone volume fraction and the bone surface area were increased (Figures 2A,C,D). Masson staining showed that the new bone and the content of collagen fibers in cancellous bone of distal femur in OVX group was significantly lower than that in Sham group. Those staining were used to observe the changes of osteogenic ability of bone tissue. After treatment with TFDG, the new bone and the content of collagen fibers in cancellous bone of distal femur in mice were significantly higher than that in OVX group, while the composition of muscle fibers was significantly decreased (Figure 2B), which suggested that the osteogenic ability was significantly improved.

### TFDG Increased the Expressions of Osteogenic Proteins in Ovariectomized Mice

**FIGURE 3:**
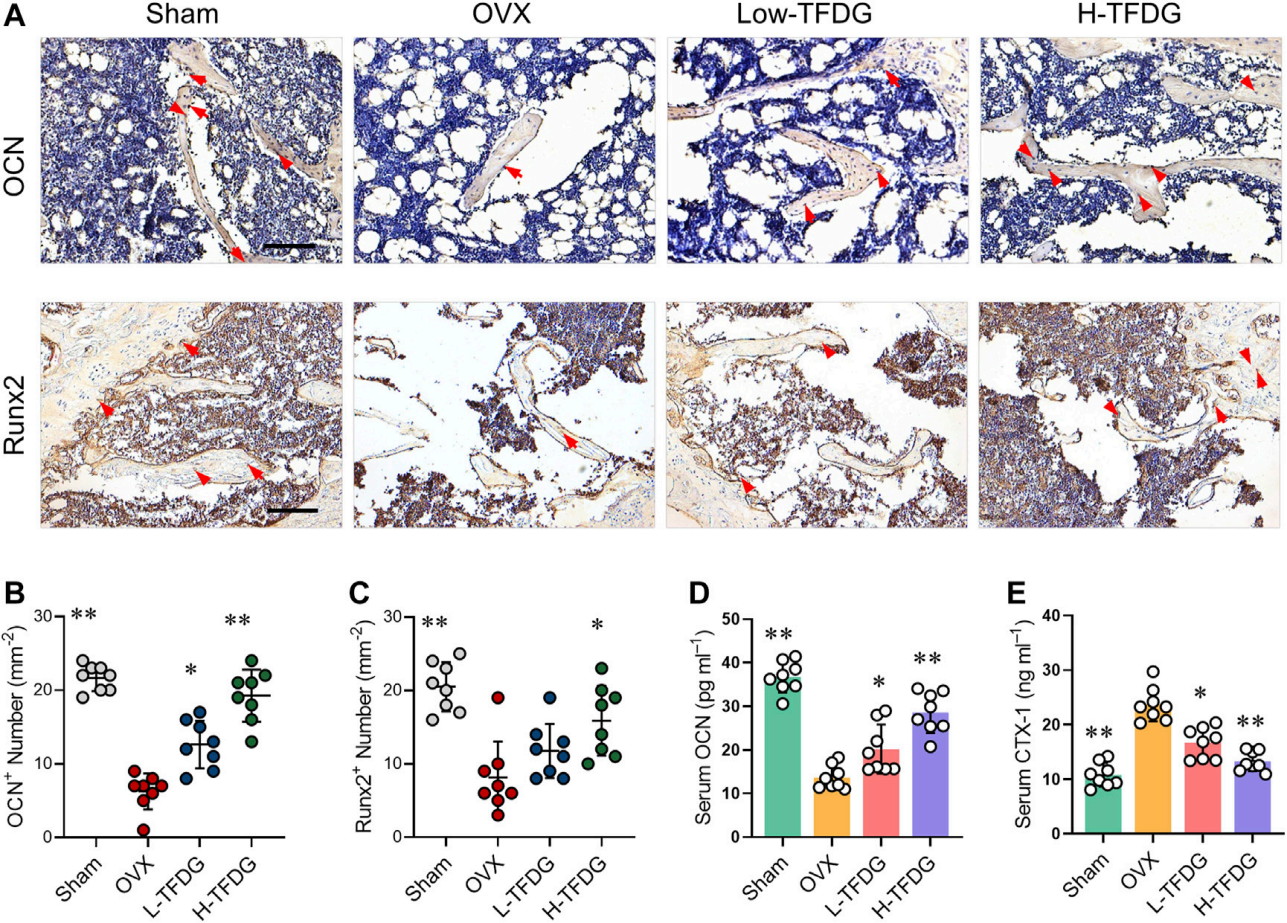
TFDG promoted the expression of osteogenic markers *in vivo*. **(A)** Representative immunohistochemical staining for OCN and Runx2 of decalcified bone sections. Scale bar = 100 μm. **(B)** Quantitative analysis of OCN positive cells and **(C)** Runx2 positive cells. **(D)** Detection of serum content of OCN and CTX-1. *n* = 8, *p < 0.05, **p < 0.01, compared with OVX group.

Immunohistochemical staining was used to detect the expression of osteocalcin OCN and osteoblast specific gene Runx2 *in vivo*. The results showed that the number of positive cells in OVX group was lower than that in Sham group. However, the number of OCN and Runx2 positive cells after TFDG treatment was higher than that in OVX group, especially in the high-dose group (Figures 3A–C). In order to explore the effect of TFDG treatment on the dynamic changes of bone formation, we collected and measured the serum levels of bone metabolism markers from mice. We measured C-terminal propeptide of type I collagen (CTX-1) and OCN, the indexes that could reflect bone resorption and bone formation. The results showed that, in OVX mice, the content of CTX-1 was higher than that in sham group mice and the content of OCN was lower. TFDG could reduce CTX-1 and increase OCN concentration at the same time, indicating that the dynamic balance of bone mass *in vivo* had changed (Figures 3D,E). These results above suggested that TFDG can improve the ability of osteogenesis and regulate the dynamic balance of bone formation in OVX mice.

### TFDG Inhibits Chronic Inflammation Induced by Ovariectomy

**FIGURE 4:**
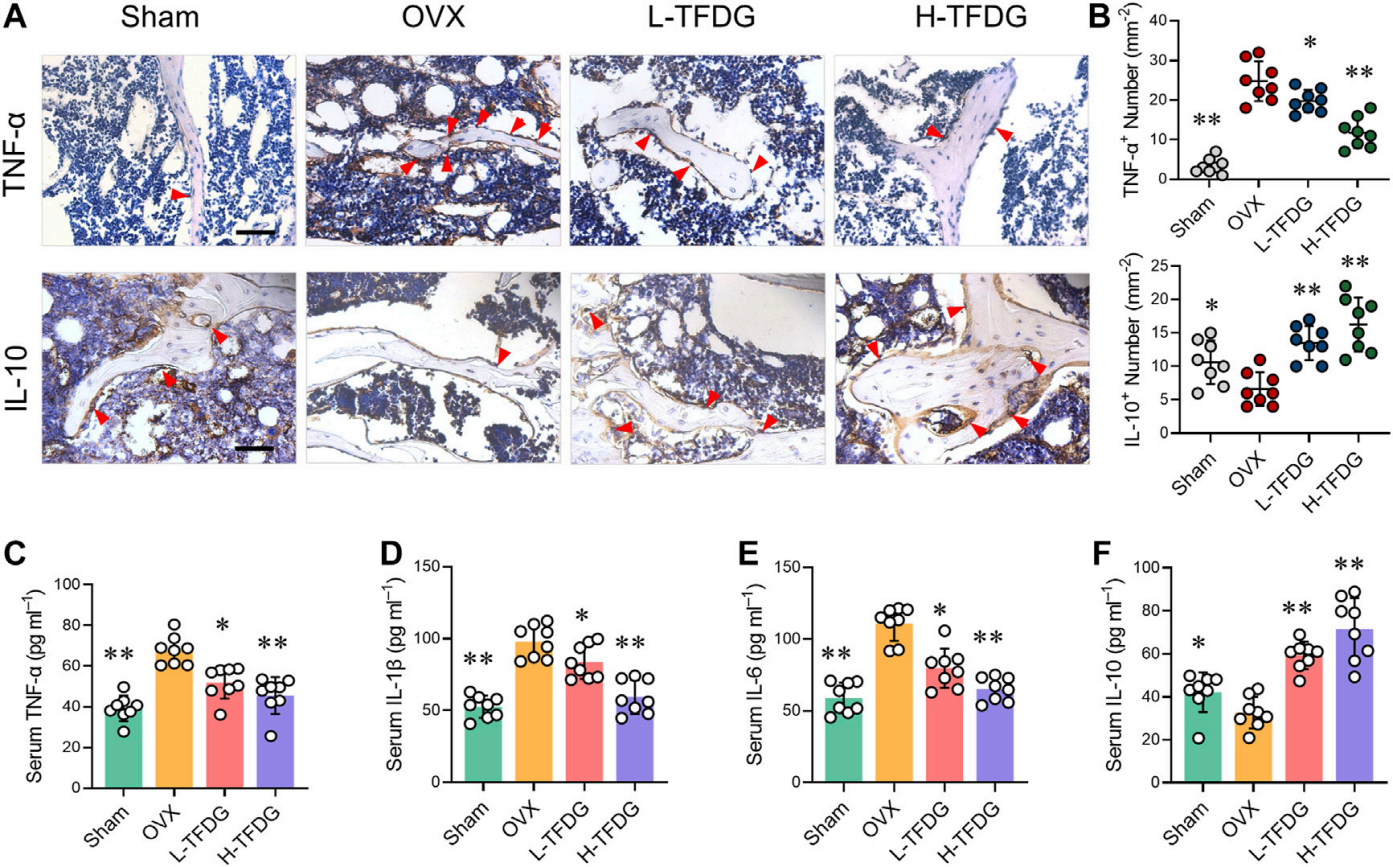
TFDG suppressed the expression of proinflammatory cytokines in OVX mice. **(A)** Representative immunohistochemical staining for TNF-α and IL-10 of decalcified bone sections. Scale bar = 100 μm. **(B)** Quantitative analysis of TNF-α positive cells and IL-10 positive cells. **(C–F)** Detection of serum content of TNF-α, IL-1β, IL-6 and IL-10. *n* = 8, *p < 0.05, **p < 0.01, compared with OVX group.

**FIGURE 5:**
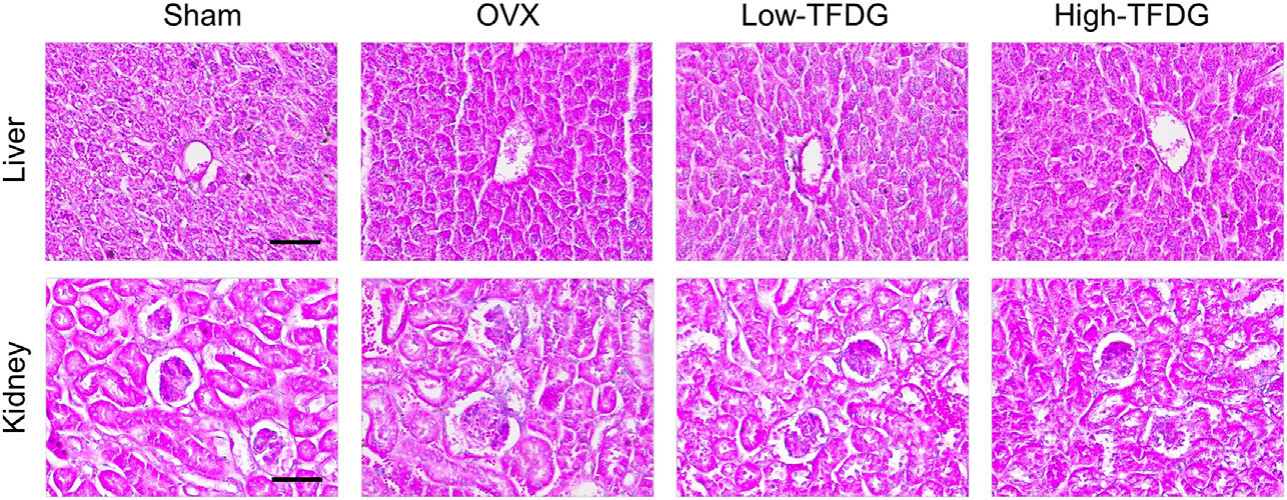
TFDG showed no toxicity on liver and kidney. Representative H&E staining of liver and kidney. Scale bar = 100 μm.

Previous studies have reported that estrogen deficiency promotes the release of proinflammatory cytokines, which makes the body present a low degree of chronic inflammation, and then inhibit osteoblast-mediated bone formation (Mundy, 2007). We found that the positive expression of proinflammatory cytokine TNF-α in trabecular area of bone in OVX mice was significantly increased by immunohistochemical staining. However, its expression was significantly decreased after TFDG treatment, and its inhibitory effect was more obvious in high dose group. On the contrary, the positive area of anti-inflammatory cytokine IL-10 in trabecular area of bone in OVX group was significantly decreased, and the expression of IL-10 was significantly increased after treatment with TFDG (Figures 4A,B). In order to further detect the systemic inflammatory state, the expressions of TNF-α, IL-1β, IL-6, and IL-10 in peripheral blood were detected by ELISA. The results showed that the expression of proinflammatory cytokines, including TNF-α, IL-1β, and IL-6, in OVX mice was significantly higher than that in sham group, while the expression of IL-10 in OVX group was slightly lower than that in sham group. However, after treatment with TFDG, the expressions of TNF-α, IL-1β, and IL-6 in TFDG treated groups were lower than those in OVX group, especially in high dose group. The anti-inflammatory cytokine IL-10 in TFDG groups was higher than that in OVX group (Figures 4C–F). There was no significant hepatorenal toxicity in all of the mice accepted *in vivo* interventions (Figure 5). The above results showed that TFDG could inhibit the expression of proinflammatory cytokines and promote the expression of anti-inflammatory cytokines in osteoporotic mice.

### TFDG Promotes Osteoblast Formation and Mineralization *In Vitro* Under Inflammatory Environment

**FIGURE 6:**
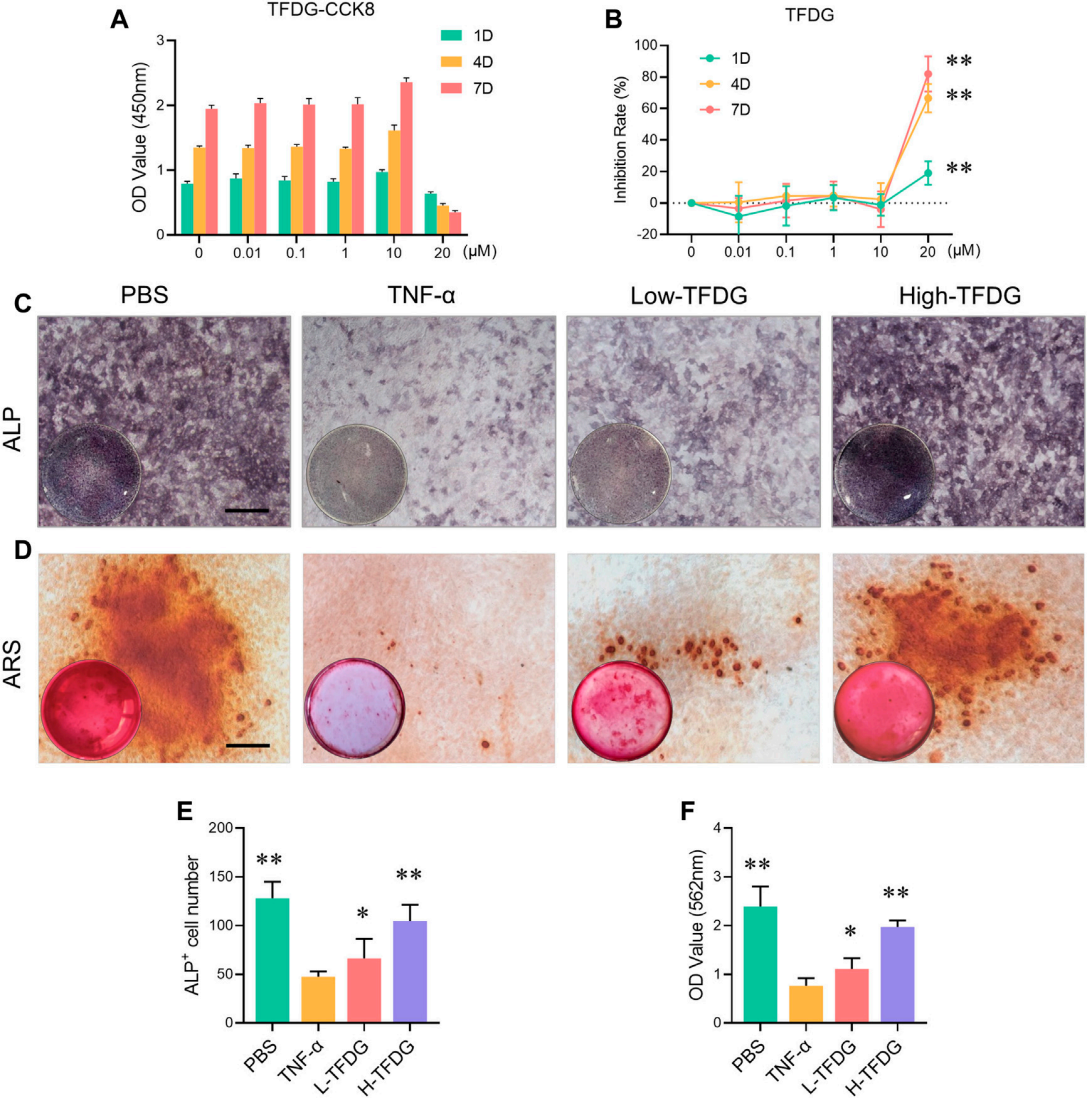
TFDG promoted osteoblast formation and mineralization in inflammatory environment. **(A)** CCK8 assay of different concentrations TFDG and different intervention time on MC3T3-E1 cells. **(B)** Inhibition rate of TFDG. *n* = 5, **p < 0.01, compared with 0 μM, 1D. **(C)** ALP staining images. MC3T3-E1 cells were cultured in osteogenic medium with or without 10 ng/ml TNF-α and different concentrations of TFDG for 7 days. Scale bar = 50 μm. **(D)** ARS staining images. MC3T3-E1 cells were cultured in osteogenic medium with or without 10 ng/ml TNF-α and different concentrations of TFDG for 25 days. Scale bar = 50 μm. **(E)** ALP positive cell number. **(F)** OD value of calcium salt absorbance. *n* = 3, *p < 0.05, **p < 0.01, compared with TNF-α group.

In order to further explore the specific mechanism of TFDG on osteoblast induction, we conducted *in vitro* osteogenic induction using MC3T3-E1 cells. CCK8 result and inhibition rate calculation suggested that TFDG had no effect on cell proliferation until the concentration reach to 20 μM (Figures 6A,B). In order to simulate inflammatory microenvironment, we added 10 ng/ml TNF-α during the induction of osteoblasts (Bu et al., 2009; Yu et al., 2019). The results of NBT/BCIP staining showed that TNF-α could significantly inhibit the alkaline phosphatase activity of MC3T3-E1 cells after osteogenic induction for 4 days. Compared with TNF-α group, ALP staining deepened with the increase of TFDG concentration in the concentration of 0.1 and 1 μM (Figure 6C). The OD value and the ALP positive cells number also decreased (Figure 6E). These results suggest that TFDG can alleviate the inhibitory effect of TNF-α on ALP activity in MC3T3-E1 cells to some extent. At the same time, the results of alizarin red staining (ARS) after 21 days of osteogenic induction showed that TNF-α could significantly inhibit the formation of calcium minerals during osteogenic induction, while the formation of calcium minerals increased after TFDG treatment, especially in high concentration group (Figure 6D). The OD value of calcium salt absorbance also proved that calcium salt was significantly increased after TFDG treatment (Figure 6F). These results might suggest that TFDG was able to promote osteoblast formation and mineralization under inflammatory environment.

### TFDG Promotes Osteoblast Protein Expression by Activating MAPK, Wnt/β-Catenin and BMP/Smad Signaling Pathways

**FIGURE 7:**
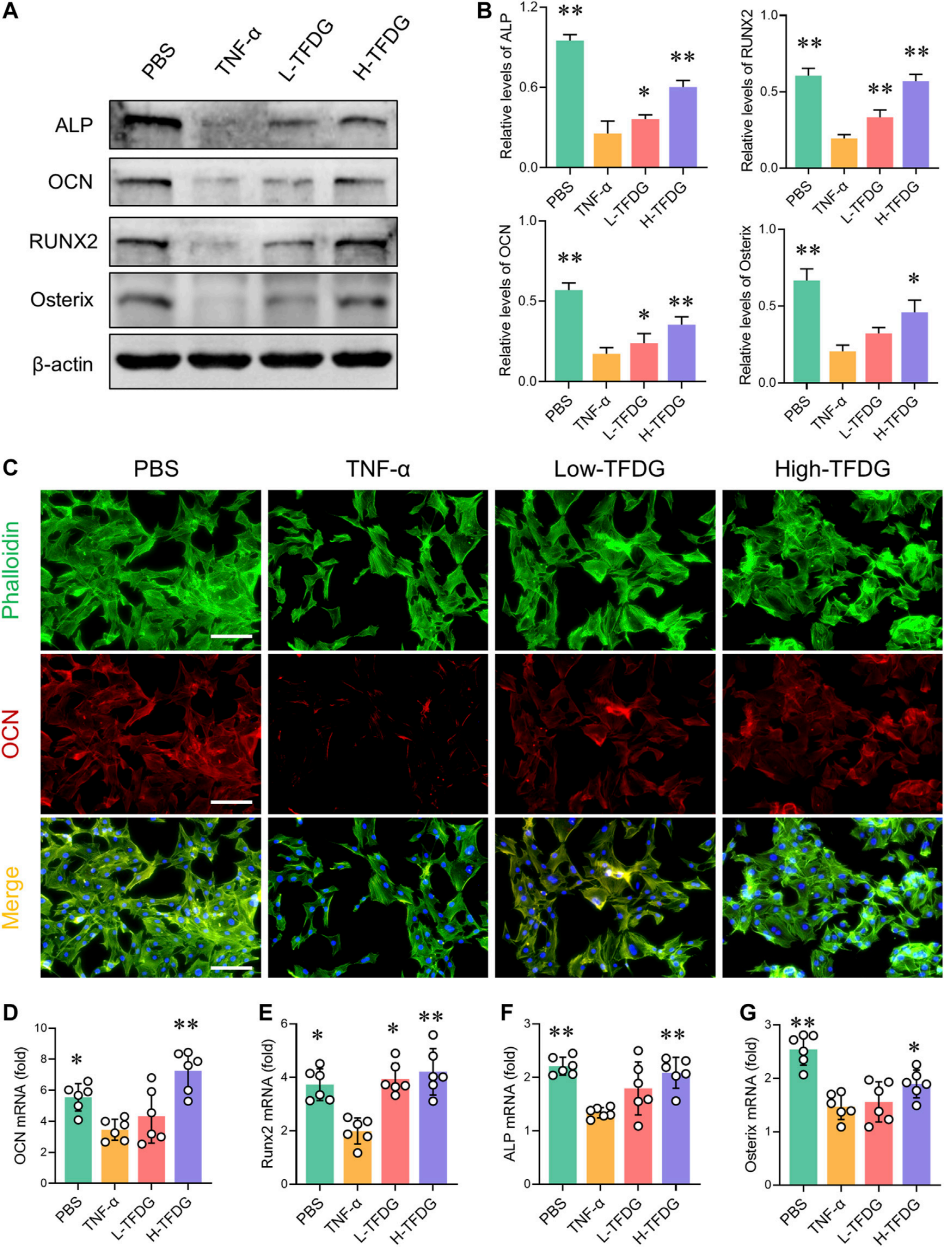
TFDG promotes the expression of osteogenic related markers under the intervention of proinflammatory cytokines. **(A)** MC3T3-E1 cells were cultured in osteogenic medium with or without 10 ng/ml TNF-α and different concentrations of TFDG (low-dose group 0.1 μM, high-dose group 1 μM) for 7 days. Western blot results and **(B)** gray value relative to β-actin. n = 3. **(C)** Immunofluorescence staining of OCN. Scale bar = 50 μm. **(D)** RT-PCR analysis of OCN, Runx2, ALP and Osterix. *n* = 6. *p < 0.05, **p < 0.01, compared with TNF-α group.

In order to detect the expressions of osteogenic related proteins, MC3T3-E1 cells were cultured with osteogenic medium under the stimulation of 10 ng/ml TNF-α and different concentrations of TFDG. Western blot results showed that after treatment with TNF-α, the expressions of ALP, OCN, Runx2, and Osterix were inhibited. However, the addition of TFDG increased expressions of these proteins within the inflammatory environment, especially in high-dose group (Figures 7A,B). By immunofluorescence staining, we could directly observe that TNF-α significantly reduced the positive expression of OCN in the process of osteogenesis induction, and the morphology of MC3T3-E1 cells under the inflammatory environment presented shrinkage when compared with the PBS treated group. The treatment of TFDG increased the number of OCN positive cells and made the cell shape more stretched (Figure 7C). The qRT-PCR analysis was used to detect the expressions of related genes during the osteogenic differentiation of MC3T3-E1 cells. We found that after being treated with TNF-α, the expressions of OCN, Runx2, ALP, and Osterix were decreased and TFDG treatment reversed the inhibition of these genes during the osteogenic differentiation (Figures 7D–G). These results suggested that TFDG was able to promote the expressions of osteogenic related proteins and genes.

**FIGURE 8:**
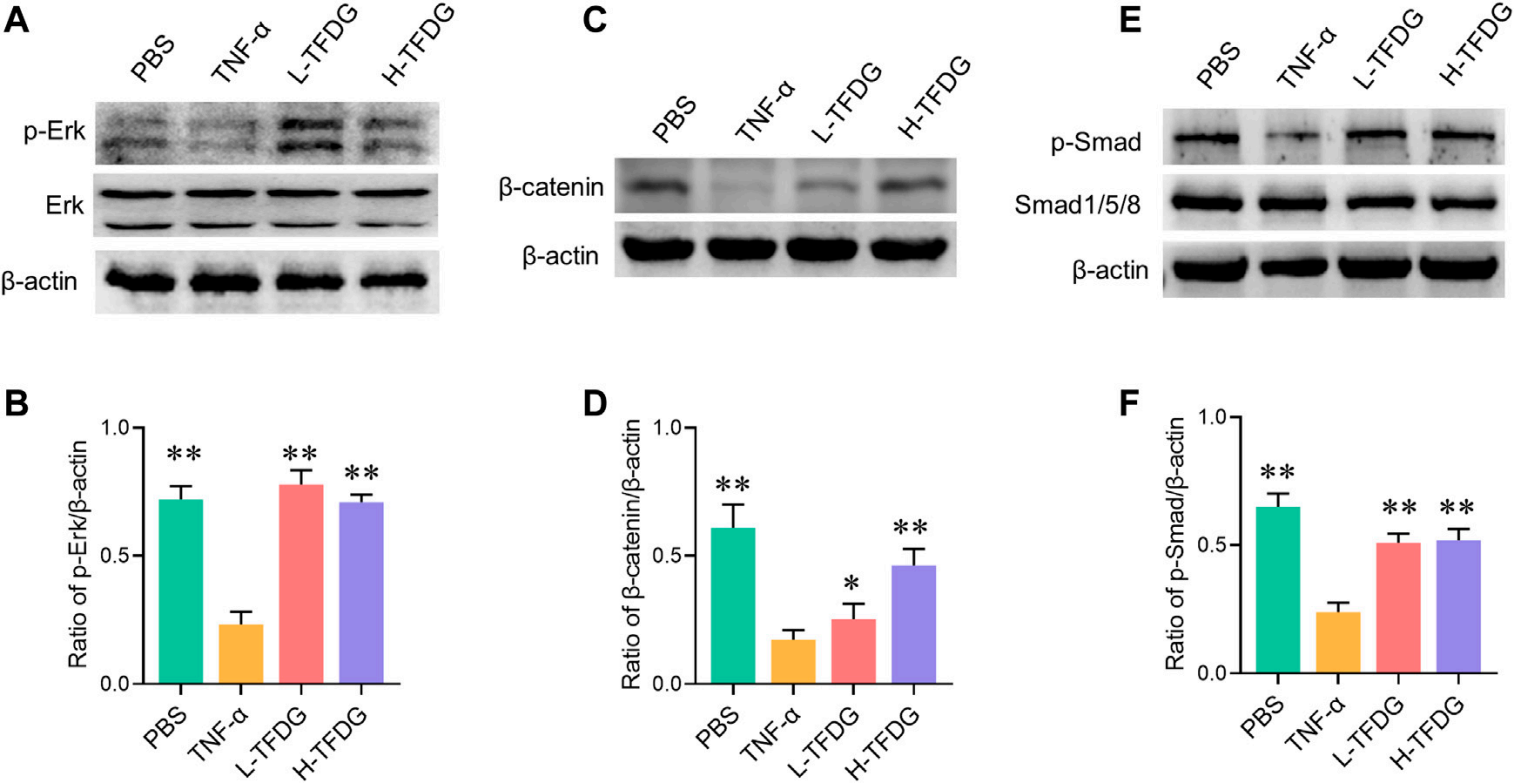
TFDG promotes the activation of osteogenic signaling pathways in inflammatory environment. **(A)** Cells were cultured in osteogenic medium with/without the intervention of 10 ng/ml TNF-α and TFDG (low-dose group 0.1 μM, high-dose group 1 μM). Western blot result of Erk and p-Erk, **(B)** gray value relative to β-actin. **(C)** Western blot result of β-catenin, **(D)** gray value relative to β-actin. **(E)** Western blot result of Smad and p-smad, **(D)** gray value relative to β-actin. n = 3, *p < 0.05, **p < 0.01, compared with TNF-α group.

During the process of osteoblast induction, MAPK, Wnt/β-catenin, and BMP/Smad signaling pathways are activated, promoting the transcription and expression of osteogenic related functional genes and proteins downstream. We further detected the key proteins in these signaling pathways to observe the activation state of these pathways. As shown in Figures 8A–F, the stimulation of TNF-α made low phosphorylation level of Erk and Smad and significant decrease in β-catenin during the process of osteoblast induction. However, this inhibited effect was weakened or even reversed after TFDG treatment. It is worth noting that the phosphorylated Erk restored high expression after intervention of low dose of TFDG, which may suggest that the MAPK signaling pathway is more sensitive to TFDG treatment.

## Discussion

Chronic inflammation associated with postmenopausal osteoporosis can promote bone destruction and damage bone formation. Many patients continue to suffer from severe bone destruction and dysfunction caused by osteoporosis. Many of the current drugs are based on anti-bone resorption. However, these may usually not be able to improve the bone mass that has been significantly lost. It has been reported that during the occurrence of osteoporosis, the ability of osteoblasts to participate in bone formation is significantly decreased, which plays an important role in bone loss (Lin et al., 2019). Therefore, how to improve the differentiation ability of osteoblasts and promote bone regeneration has become a new research direction to prevent and treat osteoporosis.

Black tea contains a variety of polyphenols, also known as catechins or tea polyphenols. TFDG is the most abundant catechin, which has a variety of biological activities and anti-inflammatory effects. Previous studies have shown that TFDG can inhibit macrophage-associated inflammatory response (Pan et al., 2000; Ukil et al., 2006). Our study also confirmed that TFDG alleviates bone loss under osteolysis condition by inhibiting osteoclast-mediated bone resorption. As a natural extract, TFDG may have less cytotoxicity and side effects compared with chemosynthetic drugs. These characteristics make TFDG a potential candidate for postmenopausal osteoporosis.

It is known that bone is a highly ordered and dynamic organ performing multiple mechanical, biological, and chemical functions. In the process of osteoporosis, the abnormality of bone metabolism is mainly caused by the decrease of bone formation and the increase of bone resorption. In Ai’s study, it reported that TFDG could reduce the activation of osteoclasts by inhibiting oxidative stress, ultimately improving osteoporosis (Ai et al., 2020). However, the effect of TFDG on bone formation is not clear. The process of bone remodeling includes the co regulation of osteoclasts and osteoblasts (Kular et al., 2012). At the resorption phase, the recruited osteoclast precursors differentiate and fuse into mature osteoclasts, and release acid phosphatase to absorb the matrix. The reversal period occurs when the rate of osteoblast differentiation is beyond osteoclast apoptosis. Finally, osteoblast mediated mineralization and new bone formation constitute the formation stage, which lasts for several months compared to the few weeks of the resorption and reversal phases (Shimada, 2013). This suggests that osteoblast mediated bone formation plays an important role in the regulation of bone homeostasis. Our study confirmed that TFDG was able to promote the expression of osteogenic signaling in inflammatory environment, enhance the differentiation ability of osteoblasts, and ultimately increase the bone mass of ovariectomized mice.

Osteoblast-mediated bone formation plays an important role in bone mass regulation. In the process of osteoporosis, the chronic inflammatory microenvironment leads to the hyperactivity of bone resorption process (Ji et al., 2016). At the same time, the formation of osteoblast is inhibited, which results in the decline of bone formation ability, and finally aggravates the changes in trabecular bone mass and microstructure, leading to the potential risk of fracture (Lee et al., 2017). By activating the function of osteoblasts, it can promote bone formation and increase bone mineral density. *In vivo* experiments, we established an OVX mouse model of osteoporosis. As expected, TFDG treatment resulted in a significant increase in bone mass and an improvement in trabecular microstructure. We found that the ovariectomized mice showed a mild inflammatory environment, and the expression of osteogenic related proteins was significantly reduced. After treatment with TFDG, the osteogenic index in bone tissue of mice returned to a normal level. This suggests that TFDG can increase bone mass loss in osteoporosis by promoting osteogenesis.

Osteoblasts are responsible for bone synthesis and mineralization (Harada and Rodan, 2003). When osteoblast precursor cells differentiate into osteoblasts, the transcription factor Runx2 is upregulated to promote the osteoblast lineage (Vimalraj et al., 2015). Studies have shown that high concentration of TNF-α can induce osteoblast apoptosis (Wang et al., 2019), while low concentration of TNF-α inhibits osteoblast differentiation and reduces the activity of ALP (Bu et al., 2009; Yu et al., 2019). This effect may explain the inhibition of bone formation in chronic inflammation. In order to further study whether and how TFDG promotes the differentiation of osteoblasts, we established an TNF-α intervention model of MC3T3-E1 cells *in vitro*. Previous studies have shown that TFDG could reduce the release of inflammatory cytokines in a variety of cells induced by LPS (Wu et al., 2017). At the same time, it also alleviated the inflammatory response induced by TNF-α in periodontitis and other diseases, which is consistent with our results (Lagha and Grenier, 2019). In the process of postmenopausal osteoporosis, the human body is in a low degree of inflammation. In our study, we used TNF-α to simulate the inflammatory microenvironment *in vitro*, and then explored the effect of TFDG on inflammation caused osteoblast inhibition. We found that TFDG not only promotes the formation of osteoblasts in inflammatory environment, but also enhances the mineralization ability of osteoblasts. At the same time, we verified that TFDG had no significant effect on the proliferation of MC3T3-E1 cells without TNF-α intervention while it had the ability to promote osteoblast differentiation under the condition of simple osteogenic induction to a certain extent (Supplementary Figures S1A–C). With the gradual enhancement of osteoblast differentiation ability, ALP, OCN and other genes are highly expressed. Runx2 and Osterix are two important transcription factors in the process of osteoblast directional differentiation, which participate in the regulation of osteoblast division cycle and promote the formation of phenotype markers of osteoblast differentiation (Han et al., 2017; Komori, 2019). TFDG treatment significantly increases the expression of these osteogenic markers under the intervention of TNF-α and promote the formation of osteoblasts eventually.

Osteoblast differentiation and bone formation are regulated by multiple signaling pathways. MAPK, Wnt/β-catenin and BMP/Smad signaling pathways are activated during osteogenesis process (Chen et al., 2012; Kim et al., 2019; Liu et al., 2019). Smad specifically binds to the activated BMP and enters the nucleus to activate osteoblast specific transcription factors including Runx2 and Osterix (Chen et al., 2012). The signal transduction of β-catenin plays an important role in the proliferation and differentiation of osteoblasts (Choi et al., 2018). We found that the activation of these signaling pathways was significantly inhibited in the process of osteogenic induction under the simulated inflammatory microenvironment. However, this inhibition effect was significantly reversed when TFDG was added as a therapeutic agent, which revealed that TFDG may be an effective drug acting on osteogenic signaling pathway.

Although we found that TFDG can significantly promote the differentiation of osteoblasts in inflammatory environment, we still need to take some other issues into consideration. In the process of osteoporosis, the abnormality of bone metabolism is mainly caused by the decrease of bone formation as well as the increase of bone resorption. In addition to the osteogenic effect, we confirmed that TFDG could reduce the number of osteoclasts around trabecular bone in ovariectomized mice and inhibit osteoclast activation *in vitro* by tartrate resistant acid phosphatase (TRAP) staining (Supplementary Figures S2A,B). The regulation of inflammatory environment is also an important part of osteoporosis research. We found that TFDG might have no significant influence on NF-κB signaling pathway during the intervention of TNF-α (Supplementary Figures S3A,B). In the future, we will further explore the regulatory mechanism of TFDG on chronic inflammation under osteoporotic condition.

## Conclusion

**FIGURE 9:**
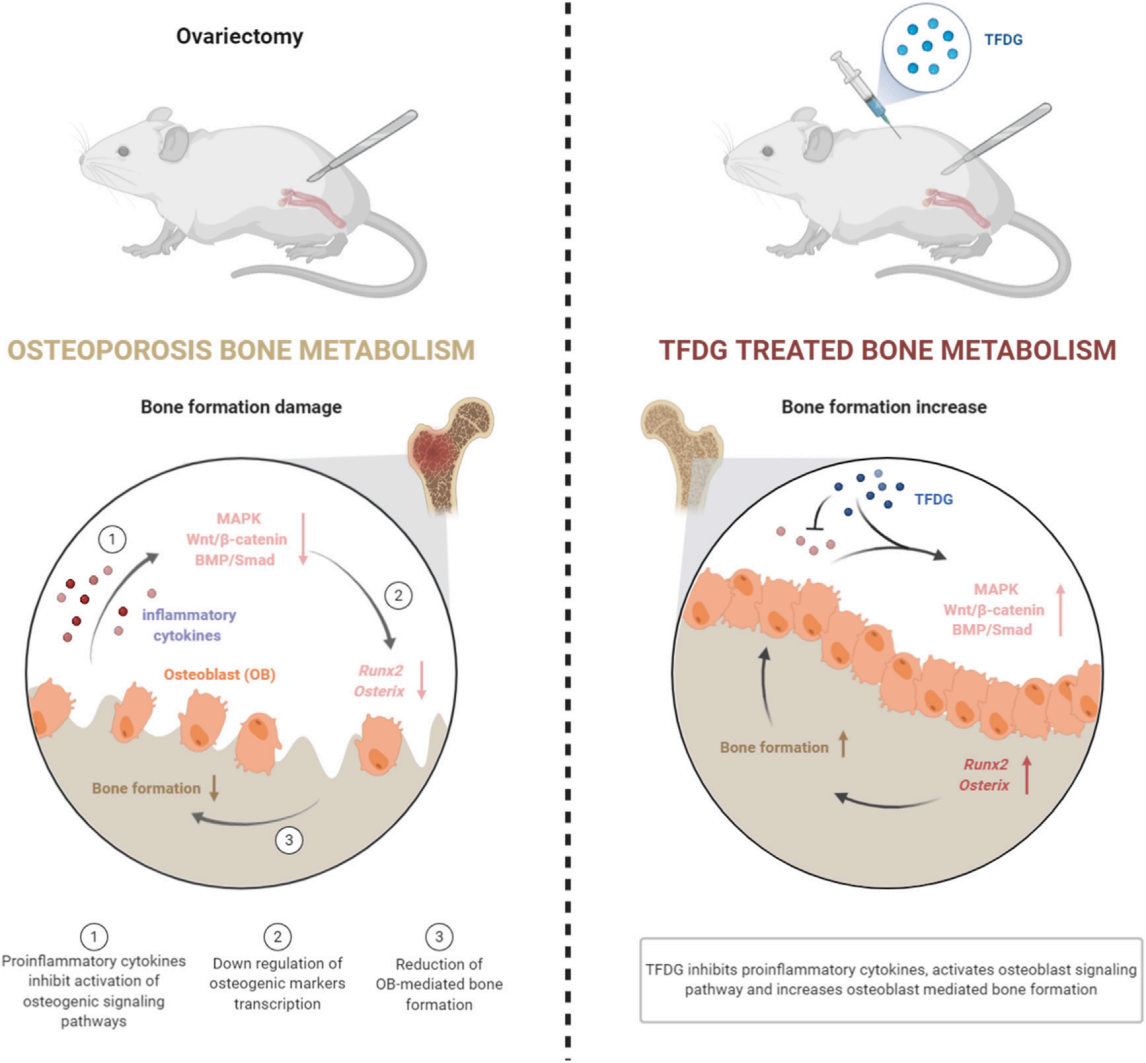
Graphic summary of TFDG promoting bone formation mechanism in OVX mice.

Our results suggest that TFDG significantly increases bone mass in ovariectomized mice by alleviating osteogenic inhibition in an inflammatory environment. Under the intervention of inflammatory cytokines, TFDG can promote the activation of osteogenic signaling pathway, enhance the transcription of osteogenic related factors, and ultimately increase the formation of osteoblasts and the ability of bone formation (Figure 9). In conclusion, we believe that TFDG can be used as a potential better drug for the prevention and treatment of postmenopausal osteoporosis.

## Data Availability

The raw data supporting the conclusions of this article will be made available by the authors, without undue reservation.
