# Supplementary material for: Theaflavin-3,3′-Digallate Promotes the Formation of Osteoblasts Under Inflammatory Environment and Increases the Bone Mass of Ovariectomized Mice
Source: Front Pharmacol. 2021 Mar 23;12:648969. doi: 10.3389/fphar.2021.648969 (PMC8021853; doi:10.3389/fphar.2021.648969)
Supplement: Supplementary file 1 [file datasheet1.docx]

**Supplementary Materials**

**Theaflavin-3,3**'**-digallate promotes the formation of osteoblasts under inflammatory environment and increases the bone mass of ovariectomized mice.**

Gaoran Ge^1^*, Sen Yang^2^*, Zhenyang Hou^3^*, Minfeng Gan^1^*, Huaqiang Tao^1^, Wei Zhang^1^, Wenming Li^1^, Zheng Wang^4^, Yuefeng Hao^5†^, Ye Gu^6†^, Dechun Geng^1†^

1: Department of Orthopaedics, The First Affiliated Hospital of Soochow University, Suzhou, 215006, China;

2: Suzhou Ninth People's Hospital Suzhou Ninth Hospital affiliated to Soochow University, Suzhou, 215006, China

3: Department of Orthopaedics, Tengzhou Hospital Affiliated to Xuzhou Medical University, Tengzhou, Shandong 277500, China;

4: Department of Orthopaedics, Suzhou Kowloon Hospital Shanghai Jiao Tong University School of Medicine, Suzhou, 215006, China;

5: Orthopedics and Sports Medicine Center, Suzhou Municipal Hospital (North District), Nanjing Medical University Affiliated Suzhou Hospital, 242, Guangji Road, Suzhou, 215006, China;

6: Changshu Hospital Affiliated to Soochow University, First People’s Hospital of Changshu City, Changshu 215500, China.

^†^**Corresponding authors:**

**Dechun Geng,** **M.D, Ph.D** E-mail: [szgengdc@163.com](mailto:szgengdc@163.com)

**Ye Gu, M.D, Ph.D** E-mail: [edwingguye@126.com](mailto:edwingguye@126.com)

**Yuefeng Hao, M.D, Ph.D** E-mail: [13913109339@163.com](mailto:13913109339@163.com)

***Contribute equally to this work**

**Supplementary Methods**

**Tartrate resistant acid phosphatase (TRAP) staining**

For *in vitro* experiments, bone marrow derived macrophages (BMM) were extracted from the femur of 8-week-old mice. BMMs were cultured in α-minimum essential medium (α-MEM) containing 10% fetal bovine serum (FBS), 30 ng/mL macrophage colony stimulating factor (M-CSF) (R&D Systems, Minnesota, USA) and 100 U/ml of Penicillin/Streptomycin (P/S, NCM Biotech, Suzhou, China). In order to induce osteoclasts, 50 ng/ml RANKL was added and cultured for 5 days. The TRAP staining of cells and tissue samples was carried out using TRAP kit (Sigma, St. Louis, Missouri, USA). The experiment was carried out according to the manufacturer’s protocols.

**Live/dead cell staining**

The MC3T3-E1 cells were cultured with or without TFDG for 7 days and a live/dead cell staining kit (AmyJet Scientific, Wuhan, China) was used for cell staining. After 30 min, the stained cells were observed under a laser confocal microscope (Leica, TCS SP8, Germany) with the dead cells stained red and the live cells green.

**Supplementary Figures**


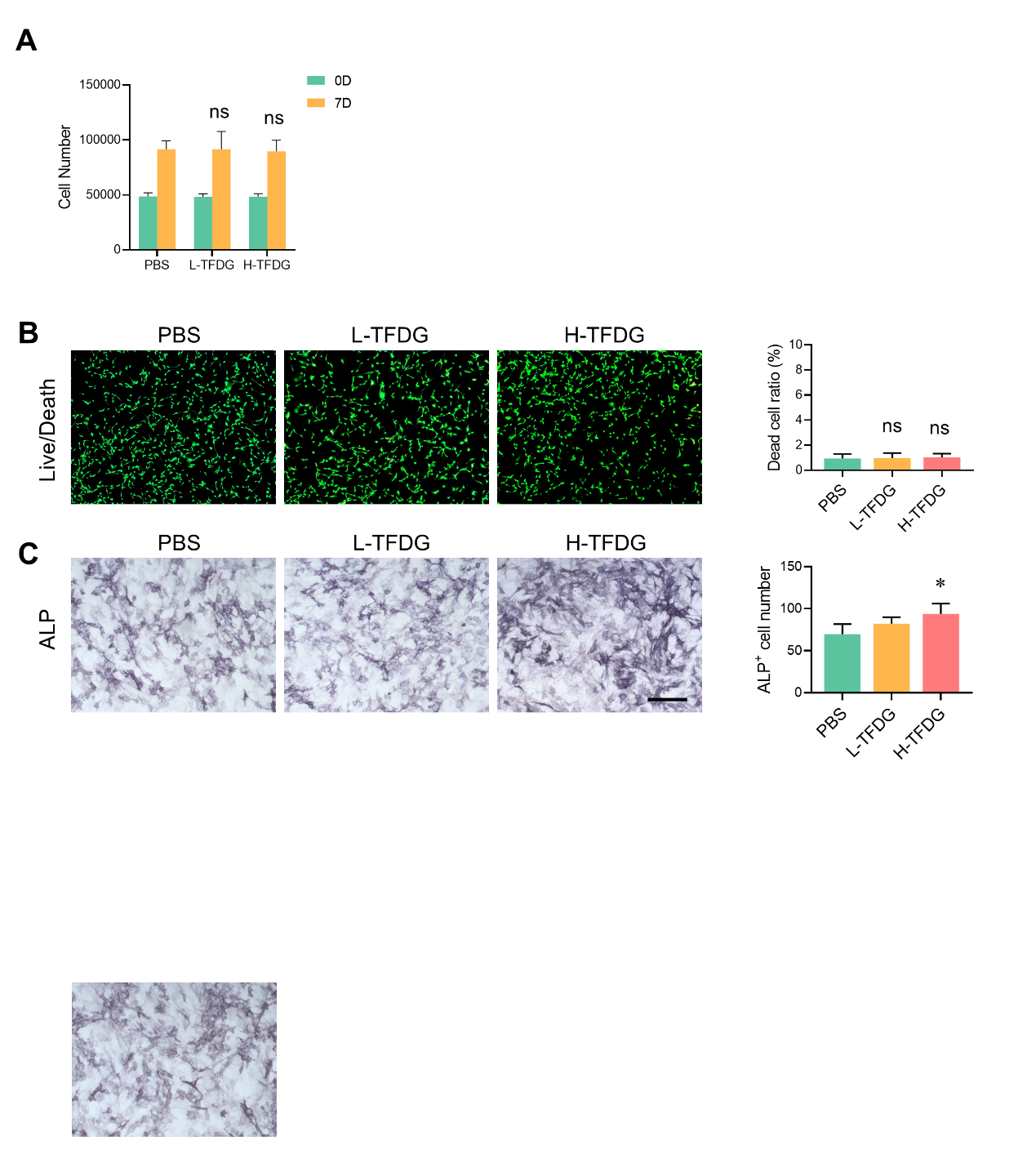


Figure S1 (A) MC3T3-E1 cells were seeded into six well plates with an initial density of 50, 000/well, and the cells were counted after 7 days (Low, 0.1 μM TFDG; High, 1 μM TFDG). (B) Live/dead staining of MC3T3-E1 cells that were cultured with or without TFDG for 7 days, and live/dead cell ratio. (C) MC3T3-E1 cells were cultured in osteogenic medium with or without 10 ng/ml TNF-α and different concentrations of TFDG for 7 days. ALP staining, and semi-quantitative analysis of the number of ALP-positive cells. Scale bars=50 μm. **p*<0.05, ns=not significant, compared with PBS group.


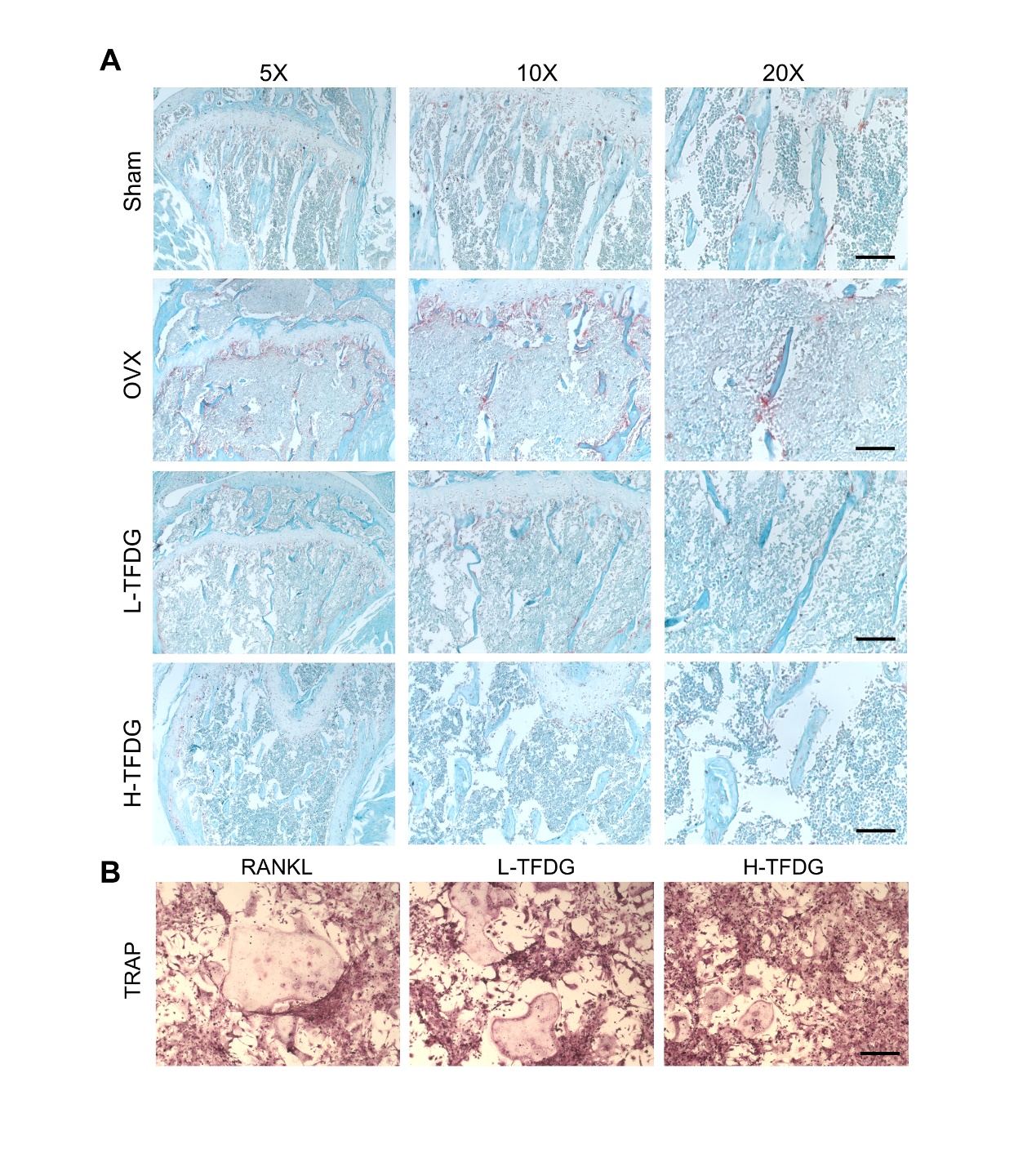


Figure S2 TFDG reduced the number of osteoclasts *in vivo* and inhibited osteoclast formation *in vitro.* (A) TRAP staining images of bone sections. Scale bars = 100 μm. (B) Bone marrow derived macrophages (BMMs) were Extracted from mouse femur. Cells were stimulated with 50 ng/ml RANKL and intervened with PUN for 4 days. TRAP staining for osteoclasts. Scale bars=50 μm.


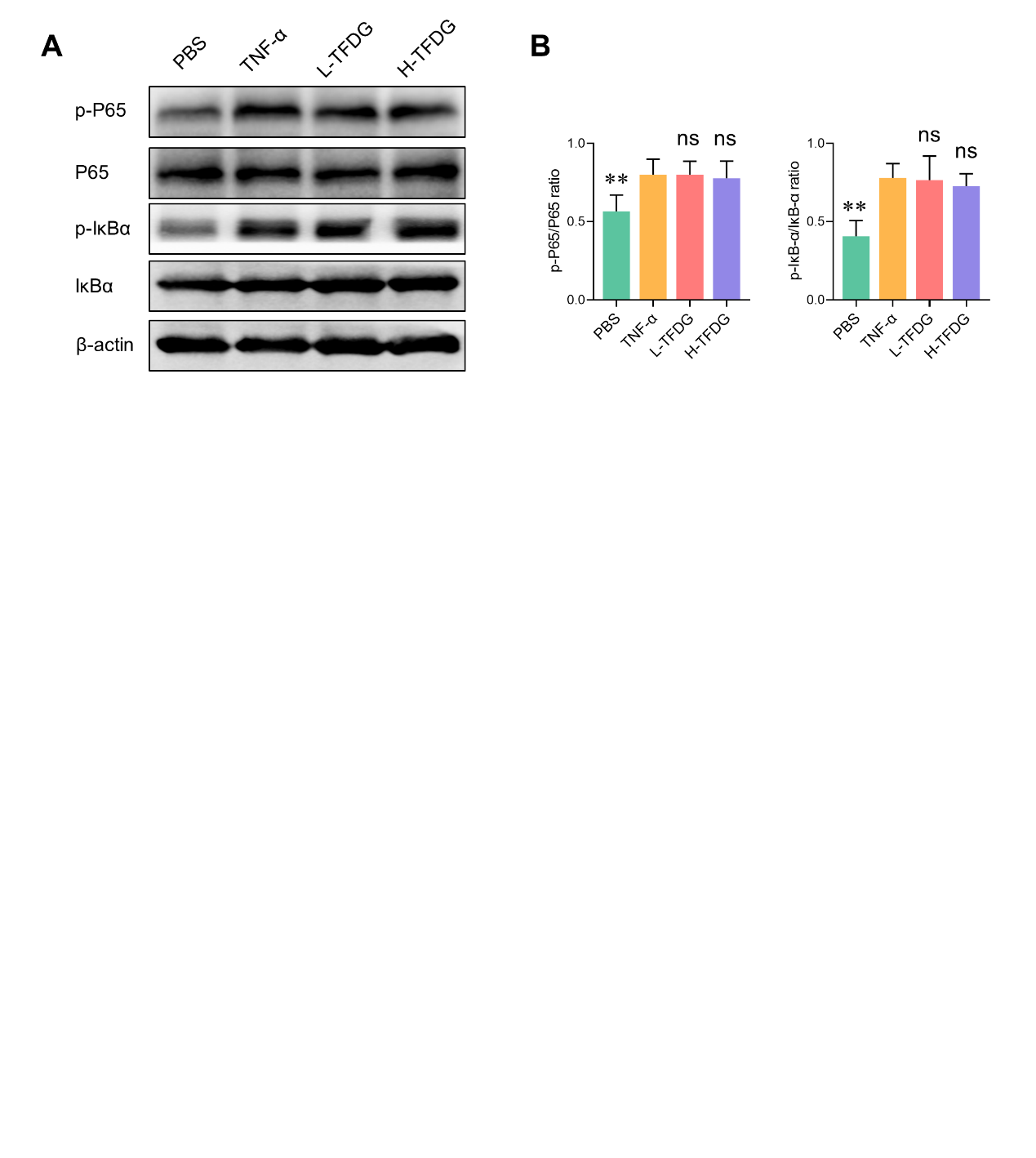


Figure S3 TFDG showed no significant effect on the activation of NF-κB signaling pathway. (A) MC3T3-E1 cells were cultured in osteogenic medium with or without 10 ng/ml TNF-α and different concentrations of TFDG (low-dose group 0.1 μM, high-dose group 1 μM) for 24 h. Western blot results and (B) gray value relative to β-actin. n=3. ***p*<0.01, ns=not significant, compared with PBS group.

**Supplementary Table**

**Table S1**

| **Primer sequence** | | |
| --- | --- | --- |
| **mRNA** | **Forward (****5**ʹ**-3**ʹ**)** | **Reverse (5**ʹ**-3**ʹ**)** |
| *ALP* | CAGCGGGTAGGAAGCAGTTTC | CCCTGCACCTCATCCCTGA |
| *OCN* | GAGGCTCTGAGAAGCATAAA | AGGGCAATAAGGTAGTGAA |
| *Osterix* | TGAGCTGGAACGTCACGTGC | TGAGCTGGAACGTCACGTGC |
| *Runx2* | GACTGTGGTTACCGTCATGGC | ACTTGGTTTTTCATAACAGCGGA |
| *GAPDH* | GGTTGTCTCCTGCGACT TCA | TGGTCCAGGGTTTCTTACTCC |
